# Supplementary material for: Improvement of immune dysregulation in individuals with long COVID at 24-months following SARS-CoV-2 infection
Source: Nat Commun. 2024 Apr 17;15:3315. doi: 10.1038/s41467-024-47720-8 (PMC11024141; doi:10.1038/s41467-024-47720-8)
Supplement: Supplementary file 1 — Supplementary Information [file 41467_2024_47720_MOESM1_ESM.pdf]

Supplementary Figure 1

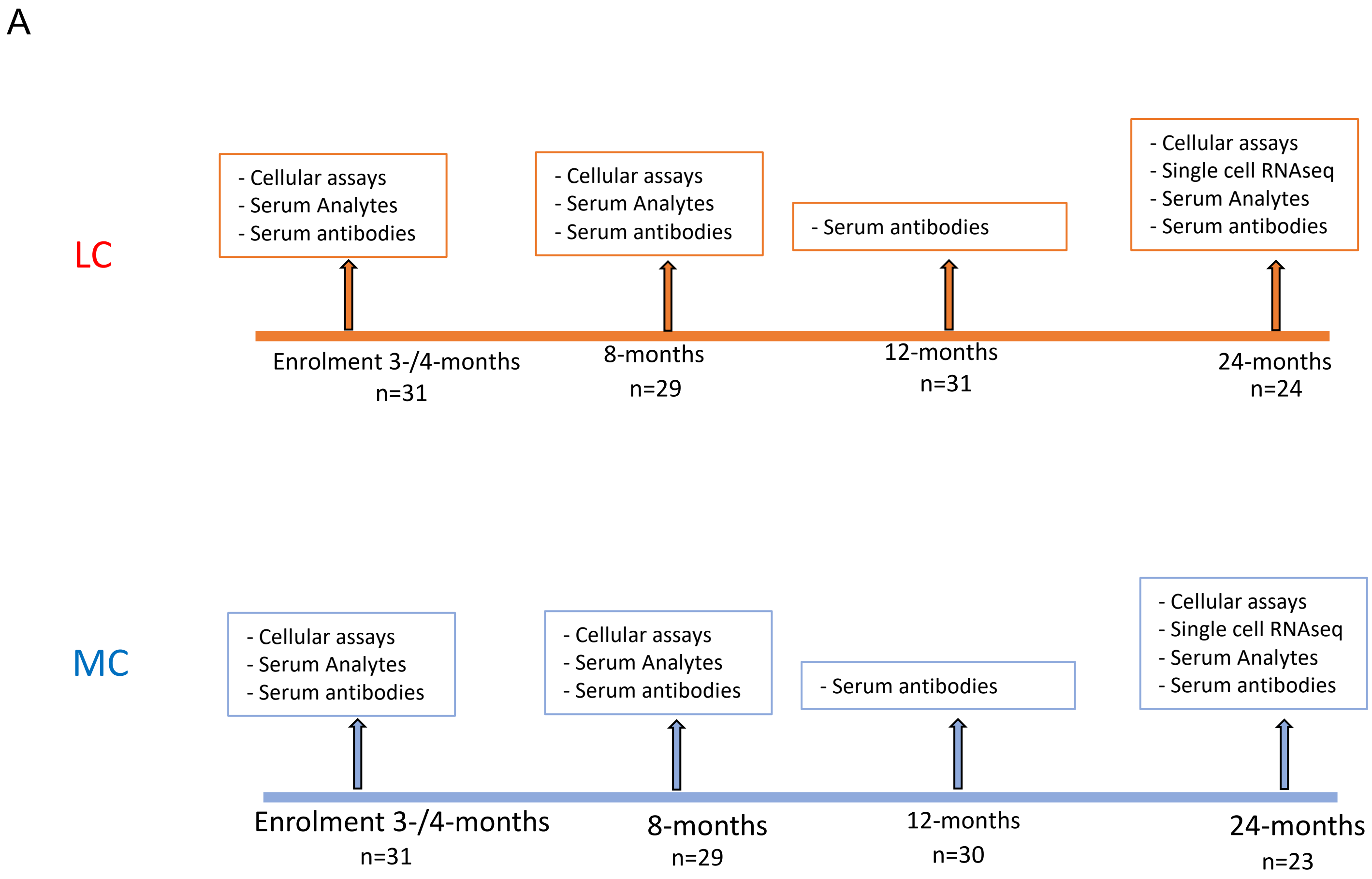

Supplementary Figure 1. *Schematic of ADAPT study.* A) Timeline detailing the number of participants and assays conducted at each timepoint. LC= Long COVID, MC= asymptomatic matched controls.

Supplementary Figure 2

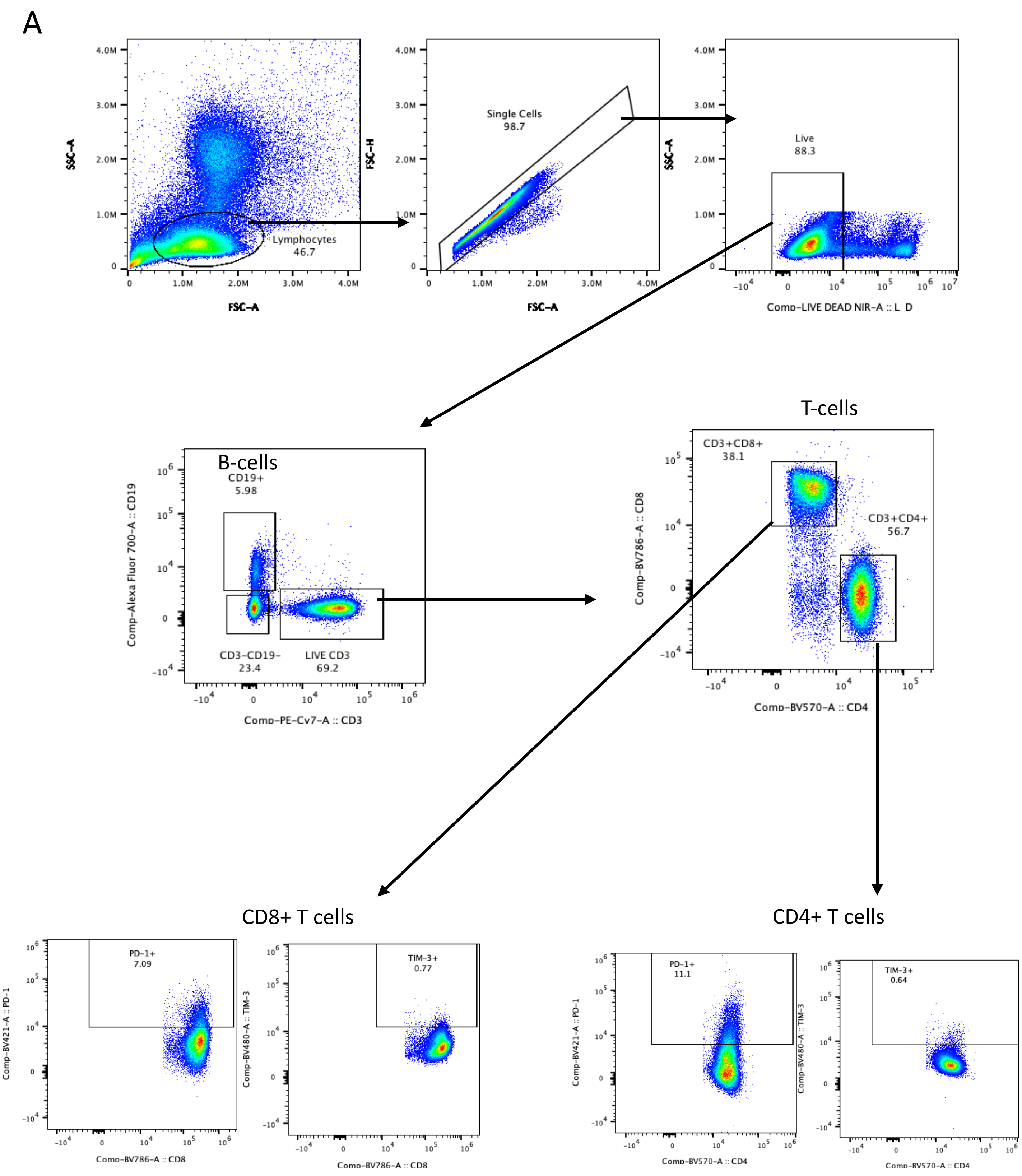

Supplementary Figure 2. *T and B cell grating strategy.* A) Representative dot plots showing gating of T and B cells and Inhibitory receptors PD-1 and Tim-3.

Supplementary Figure 3

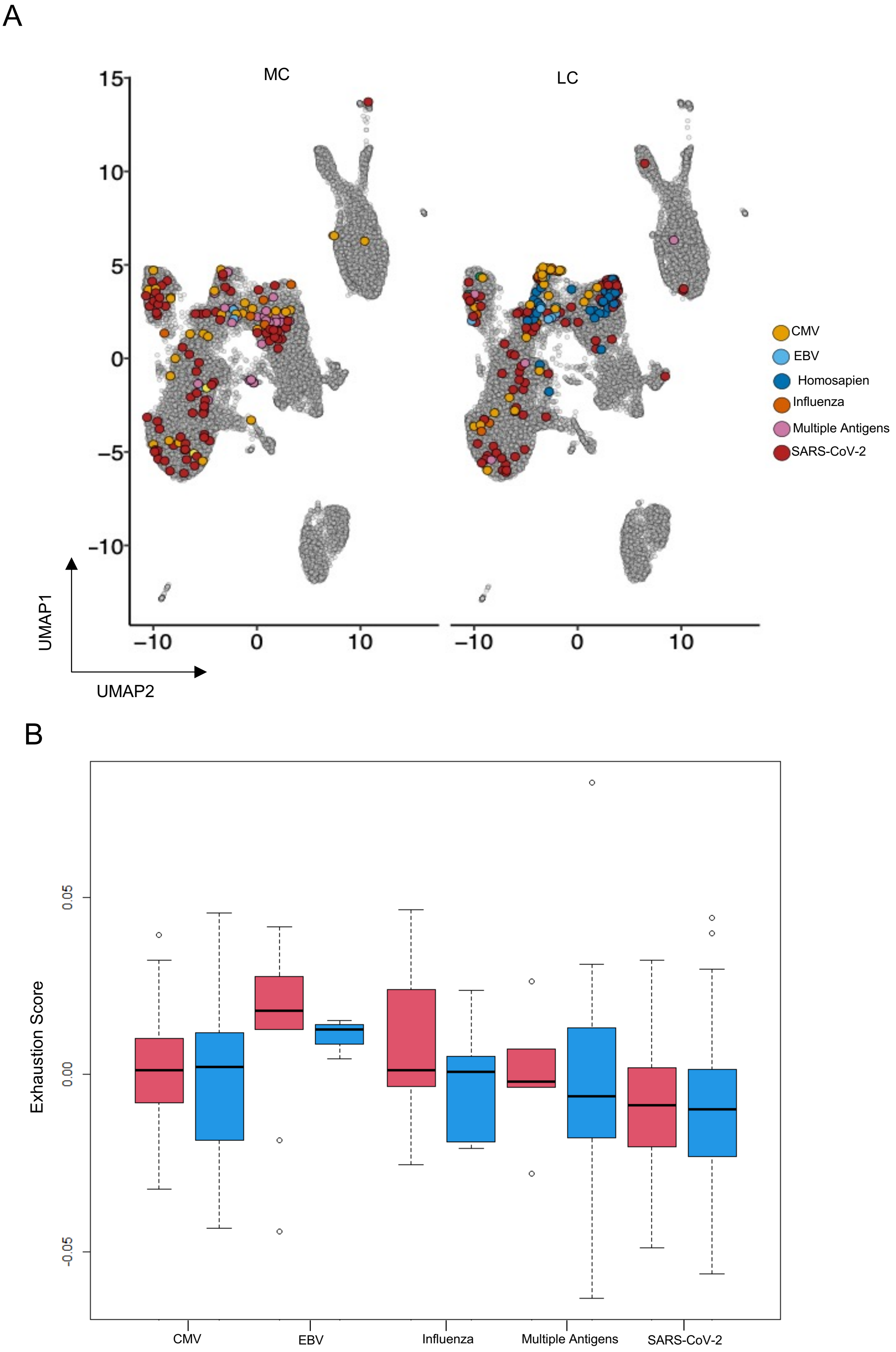

Supplementary Figure 3. *Exhaustion scores in antigen-specific T cell clones.* A) T cell clones identified from single-cell RNAseq data were overlayed on UMAP showing paired TCRS mapped to specific antigens. B) No difference between exhaustion scores in T cells specific to varying antigens including SARS-CoV-2. Red bars= Long COVID, blue bars= asymptomatic matched controls.

Supplementary Figure 4

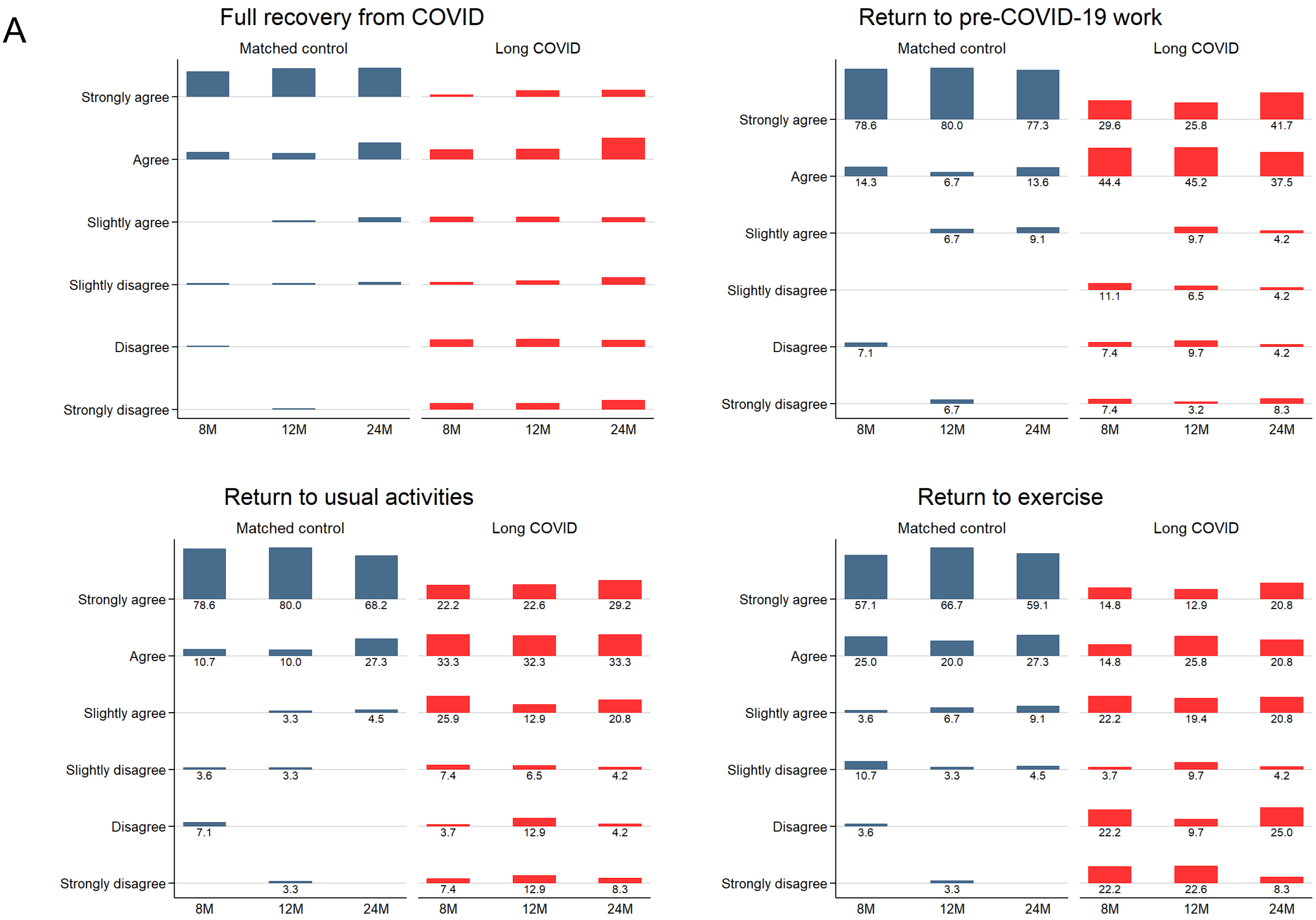

Supplementary Figure 4. *Changes to functional status over time* . A) Percent of participants from LC and MC responding to functional queries regarding full recovery from COVID-19, return to pre-COVID work, return to usual daily living, and return to normal exercise level assessed at 8-, 12- and 24-months, n=31 (LC) and n=31 (MC).

Supplementary Figure 5

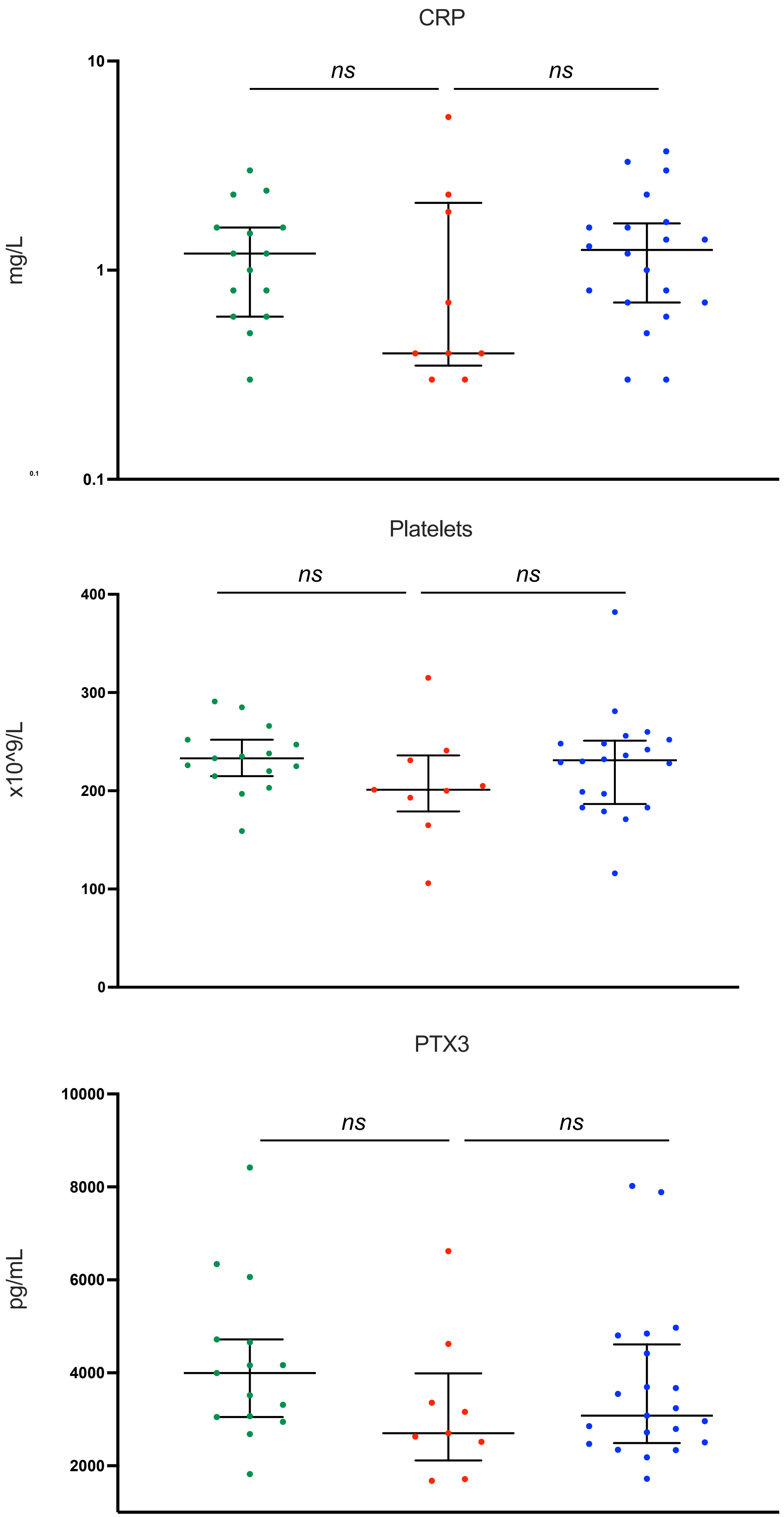

Supplementary Figure 5. *Blood biomarkers at 24-months. A) Dot plots showing concentrations of CRP, Platelets and PTX 3 at 24-months; recovered (green), un-recovered (red), matched controls (blue).*
