# Supplementary material for: Improvement of immune dysregulation in individuals with long COVID at 24-months following SARS-CoV-2 infection
Source: Nat Commun. 2024 Apr 17;15:3315. doi: 10.1038/s41467-024-47720-8 (PMC11024141; doi:10.1038/s41467-024-47720-8)
Supplement: Supplementary file 3 — Reporting Summary [file 41467_2024_47720_MOESM3_ESM.pdf]

nature portfolio

Corresponding author(s): Chansavath Phetsouphanh
Anthony Kelleher
Gail Matthews

Last updated by author(s): March 18, 2024

Reporting Summary

Nature Portfolio wishes to improve the reproducibility of the work that we publish. This form provides structure for consistency and transparency in reporting. For further information on Nature Portfolio policies, see our Editorial Policies and the Editorial Policy Checklist.

Statistics

For all statistical analyses, confirm that the following items are present in the figure legend, table legend, main text, or Methods section.

n/a	Confirmed	
☐	☒	The exact sample size (*n*) for each experimental group/condition, given as a discrete number and unit of measurement
☐	☒	A statement on whether measurements were taken from distinct samples or whether the same sample was measured repeatedly
☐	☒	The statistical test(s) used AND whether they are one- or two-sided<br>*Only common tests should be described solely by name; describe more complex techniques in the Methods section.*
☐	☒	A description of all covariates tested
☐	☒	A description of any assumptions or corrections, such as tests of normality and adjustment for multiple comparisons
☐	☒	A full description of the statistical parameters including central tendency (e.g. means) or other basic estimates (e.g. regression coefficient) AND variation (e.g. standard deviation) or associated estimates of uncertainty (e.g. confidence intervals)
☐	☒	For null hypothesis testing, the test statistic (e.g. *F*, *t*, *r*) with confidence intervals, effect sizes, degrees of freedom and *P* value noted<br>*Give P values as exact values whenever suitable.*
☒	☐	For Bayesian analysis, information on the choice of priors and Markov chain Monte Carlo settings
☐	☒	For hierarchical and complex designs, identification of the appropriate level for tests and full reporting of outcomes
☒	☐	Estimates of effect sizes (e.g. Cohen's *d*, Pearson's *r*), indicating how they were calculated

Our web collection on statistics for biologists contains articles on many of the points above.

Software and code

Policy information about availability of computer code

Data collection	All clinical data were stored using REDCap electronic data capture tools and held at St Vincents' Hospital Sydney Australia. Flow cytometry data were collected using Spectroflo v3.0 (Cytek) and FACS DIVA v9.0 (BD Biosciences).
Data analysis	Flow cytometry data analysis was performed using FlowJo version 10.7.1 (BD Biosciences). Statistical analysis was done using Prism 10 (GraphicPad, La Jolla, CA, USA) and clinica data were analysed using Stata v14 (StataCorp LLC, College Station, TX, USA). scRNA seq data analysis used R (version 4.3.0) within RStudio IDE (version 2023.3.1.446) . Code details are outlined in methods sections of manuscript.

For manuscripts utilizing custom algorithms or software that are central to the research but not yet described in published literature, software must be made available to editors and reviewers. We strongly encourage code deposition in a community repository (e.g. GitHub). See the Nature Portfolio guidelines for submitting code & software for further information.

Data

Policy information about availability of data

All manuscripts must include a data availability statement. This statement should provide the following information, where applicable:

- Accession codes, unique identifiers, or web links for publicly available datasets
- A description of any restrictions on data availability
- For clinical datasets or third party data, please ensure that the statement adheres to our policy

To protect patient privacy, underlying electronic health records may be accessed via a remote server pending a material transfer agreement. To protect patient privacy, underlying electronic health records may be accessed via a remote server pending a material transfer agreement. To access all other data please contact

corresponding authors. Study steering committee meet quarterly and will decide on data requests, CDA or collaboration agreements may be required prior to data release.

Research involving human participants, their data, or biological material

Policy information about studies with human participants or human data. See also policy information about sex, gender (identity/presentation), and sexual orientation and race, ethnicity and racism.

Reporting on sex and gender	Gender was included in the original study design. Donors from both groups were age and gender matched. Both groups contained 52% female and 48% male participants.
Reporting on race, ethnicity, or other socially relevant groupings	Ethnicity was group as either Caucasian or other than Caucasian. Caucasian being the predominant ethnic group in this study (~87%).
Population characteristics	The ADAPT study is a prospective cohort study of post-COVID-19 recovery established in April 2020. 147 participants with confirmed SARS-CoV-2 infection were enrolled, the majority following testing in community based clinics run by St Vincent's Hospital Sydney, with some self-reffered long COVID care patients also also enrolled. Initial study follow-up was planned for 12 months post-COVID-19, but was recently extended to 2 years. Extensive clinical data and a biorepository was systematically collected prospectively.
Recruitment	Donors were recruited through St Vincent's Hospital Sydney
Ethics oversight	The ADAPT study was approved by the St Vincent's Hospital, Sydney Human Research Ethics Committee (2020/ETH00964) and is a registered trial (ACTRN12620000554965). ADAPT-C sub study was approved by the same committee (2020/ETH01429). All data were stored using REDCap electronic data capture tools. Unexposed healthy donors were recruited through St Vincent's Hospital and was approved by St Vincent's Hospital, Sydney Human Research Ethics Committee (HREC/13/SVH/145).

Note that full information on the approval of the study protocol must also be provided in the manuscript.

Field-specific reporting

Please select the one below that is the best fit for your research. If you are not sure, read the appropriate sections before making your selection.

[x] Life sciences [ ] Behavioural & social sciences [ ] Ecological, evolutionary & environmental sciences

For a reference copy of the document with all sections, see nature.com/documents/nr-reporting-summary-flat.pdf

Life sciences study design

All studies must disclose on these points even when the disclosure is negative.

Sample size	Due to the low number of community acquired COVID-19 cases in Sydney (NSW, Australia) we were limited to the sample size for our study. A total of 147 participants were recruited for the ADAPT study with n=31 define has having long COVID.
Data exclusions	None
Replication	We included data on all available patients/samples. Serum analytes were done in duplicate and phenotyping by FACS were done on all available PBMC vials from donors for the 2 groups. scRNAseq was done on 20 donors, cell encapsulation and library prep was done in duplicate for each pooled sample. All work were done on human samples with limited cell availability. Attempts at replication were successful.
Randomization	Samples from the ADAPT study were grouped into Long COVID and Asymptomatic Matched controls. Patients were defined as 'long COVID' at 4-months based on the presence of any ≥ 1 of the following; fatigue, dyspnoea or chest pain 23. These patients were gender and age (+/- 10 years) matched with ADAPT participants without long COVID (Matched ADAPT controls).
Blinding	Experimental investigators for B cell tetramer work were blinded to group allocation. Blinding for other experiments were not possible as LC and MC groups were known from previous published work DOI https://doi.org/10.1038/s41590-021-01113-x

Reporting for specific materials, systems and methods

We require information from authors about some types of materials, experimental systems and methods used in many studies. Here, indicate whether each material, system or method listed is relevant to your study. If you are not sure if a list item applies to your research, read the appropriate section before selecting a response.

Materials & experimental systems

n/a	Involved in the study
[ ]	[x] Antibodies
[ ]	[x] Eukaryotic cell lines
[x]	[ ] Palaeontology and archaeology
[x]	[ ] Animals and other organisms
[ ]	[x] Clinical data
[x]	[ ] Dual use research of concern
[x]	[ ] Plants

Methods

n/a	Involved in the study
[x]	[ ] ChIP-seq
[ ]	[x] Flow cytometry
[x]	[ ] MRI-based neuroimaging

Antibodies

Antibodies used

Extracellular panel included: Live/Dead dye Near InfraRed, CD38 (HIT2, #MHCD3819) (ThermoFisher Scientific, USA); CD3 (UCHT1, # 300430), CD8 (HIL-72021, # 301042), CD123 (6H6, #306043), PD-1 (EH12.1, #562516), TIM-3 (TD3, #746771), CD27 (L128, #563167), CD45RA (HI100, #564552), IgD (IA6-2, #561315), (2A3, #340939), and CD19 (HIB19, #557921) (BioLegend, USA); CD4 (OKT4, 300533), CD127 (A019D5, #351325), HLA-DR (L234, #307671), CCR7 (G043H7, 353217), CD16 (GB11, #302054), CD14 (HCD14, #325631), CD56 (NCAM-1, #557747), CD11c (B-ly6, #561355), and CD57 (QA17A04, #393304) (BD Biosciences, USA).

In vitro activation mAb panel included: CD3 (UCHT1, # 300429), CD4 (RPA-T4, # 557922), CD8 (RPA-T8, # 301041), CD39 (A1, # 328205), CD69 (FN50, # 310911), CD137 (4B4-1, # 309820) all BioLegend, CD25 (2A3, #340939), CD134 (L106, # 340420)- BD Biosciences.

Spike probes, CD19-ECD (J3-119, #IM2708U) (Beckman Coulter), IgD AF488 (polyclonal) (#2030-30) (Southern Biotech), IgG-BV786 (G18-145, #564230), CD21-BUV737 (B-ly4, #564437), CD38 AF700 (HIT2 #560676), Streptavidin BV510 (#563261) (BD Biosciences), CD14-BV510 (M5E2, #301841), CD3 BV510 (OKT3, #317332), CD8a-BV510 (RPA-T8, #301048), CD16-BV510 (3G8, #302048), CD10-BV510 (HI10a, #312220), CD20 APC-Cy7 (2H7, #302314), CD27-BV605 (O323, #302829),CD71 PE Cy7 (CY1G4 #334112)(BioLegend).

Validation

Primary antibodies were validated and titrated with appropriate positive and negative controls. FMO and endogenous expression were used to set gates. Antibody validation by the manufacturer is available at each manufacturer's website by searching under the provided antibody part numbers.

Eukaryotic cell lines

Policy information about cell lines and Sex and Gender in Research

Cell line source(s)

State the source of each cell line used and the sex of all primary cell lines and cells derived from human participants or vertebrate models.

Authentication

Describe the authentication procedures for each cell line used OR declare that none of the cell lines used were authenticated.

Mycoplasma contamination

Confirm that all cell lines tested negative for mycoplasma contamination OR describe the results of the testing for mycoplasma contamination OR declare that the cell lines were not tested for mycoplasma contamination.

Commonly misidentified lines
(See ICLAC register)

Name any commonly misidentified cell lines used in the study and provide a rationale for their use.

Clinical data

Policy information about clinical studies
All manuscripts should comply with the ICMJEguidelines for publication of clinical research and a completedCONSORT checklist must be included with all submissions.

Clinical trial registration

ACTRN12620000554965

Study protocol

Full protocol can be provided upon request.

Data collection

Data collection started March 2020 and is currently ongoing. All data were stored using REDCap electronic data capture tools.

Outcomes

The aims of ADAPT are to evaluate a number of outcomes after COVID-19 relating to pathophysiology, immunology and clinical sequalae. Initial study follow-up was planned for 12 months post-COVID-19, but was recently extended to 2 years. All patients are now in the convalescents stage.

Flow Cytometry

Plots

Confirm that:

- [x] The axis labels state the marker and fluorochrome used (e.g. CD4-FITC).
- [x] The axis scales are clearly visible. Include numbers along axes only for bottom left plot of group (a 'group' is an analysis of identical markers).
- [x] All plots are contour plots with outliers or pseudocolor plots.
- [x] A numerical value for number of cells or percentage (with statistics) is provided.

Methodology

Sample preparation	Blood was collected for biomarker analysis (SST 8.5mLs x 1 (Serum) and EDTA 10mLs x 1 (Plasma)) and 36mLs was collected for PBMCs (ACD 9mLs x4). Phenotyping of PBMC was performed as described previously 25. Briefly, cryopreserved PBMCs were thawed using RPMI (+L-glut) medium (ThermoFisher Scientific, USA) supplemented with Penicillin/Streptomycin (Sigma-Aldrich, USW), and subsequently stained with antibodies binding to extracellular markers.
Instrument	Samples were acquired on an Cytek Aurora (Biolegend, USA) and BD LSR Fortessa (USA).
Software	Spectroflo and FACS Diva software was used for Sample acquisition and FlowJo was used for analysis.
Cell population abundance	No cell sorting experiments were performed.
Gating strategy	Provided in previous publication as Extended data figure 6, doi https://doi.org/10.1038/s41590-021-01113-x

- [ ] Tick this box to confirm that a figure exemplifying the gating strategy is provided in the Supplementary Information.
